# Medical Students’ Experiences of a National Student Psychiatry Conference: A Mixed-Methods Evaluation

**DOI:** 10.1192/bjo.2026.11308

**Published:** 2026-06-30

**Authors:** Joseph Kendall, Su Ying Yeoh, Sophie Krug, Keiru Ajuzieogu, Joanne Rodda, Ella Haines, Priscilla Yowa, Ramneek Kaur, Naomi King, Doreen Kuek, Alexander Lawrence, Jamie Mawer, Swara Rajurkar, Sanem Ucur, Leera Vite, Sukhi Shergill

**Affiliations:** 1 Kent and Medway Medical School, Canterbury, United Kingdom; 2 Kent and Medway Mental Health NHS Trust, Maidstone, United Kingdom

## Abstract

**Aims:**

The National Psychiatry Student Conference (NPSC) is an annual student-led conference supported by the RCPsych Choose Psychiatry programme. The 2026 NSPC was hosted by Kent and Medway Medical School. Understanding student perspectives of such enrichment activities is important to inform future conference design and maximise educational impact. This mixed-methods study aimed to examine students’ perceptions of thequality, relevance and impact of the conference, including attitudes towards psychiatry and future career intentions.

**Methods:**

All student delegates attending the 2026 NSPC were invited to complete an anonymous post-conference questionnaire including Likert-scale items and free-text responses exploring overall experience, session quality and perceived impact. A purposive sample of respondents was invited to take part in semi-structured interviews to explore experiences in greater depth. Questionnaire data were analysed descriptively, and interview data were analysed using thematic analysis.

**Results:**

Of 103 delegates, 68 students completed the questionnaire and 11 were interviewed.

Questionnaire data indicated positive experiences of the conference overall (98% were satisfied or very satisfied), and of the individual programmed activities, with an average session score of 4.5/5. The most popular sessions included a consultant’s journey through their career and lived experience of mental illness, functional neurological disorder, interactive workshops, and a subspeciality panel Q&A. Ninety percent of respondents reported that the conference had increased their interest in psychiatry as a career.

Interview data aligned closely with these findings. Students described the conference as well organised, welcoming and engaging, and valued opportunities to hear directly from clinicians about career pathways and the breadth of psychiatric practice. Many reported that the conference challenged preconceptions about psychiatry and increased awareness of the range of subspecialties and research opportunities.

For many delegates, this was their first academic conference. Barriers to attendance included travel distance and cost, with subsidised accommodation and low-cost tickets described as important enablers of participation.

**Conclusion:**

This mixed-methods evaluation demonstrates that a student-led national psychiatry conference was experienced as engaging and educationally valuable by attending, self-selecting students, with delegates reporting more positive attitudes towards psychiatry as a career and a deeper understanding of the specialty. Findings also highlighted practical barriers to participation, particularly cost and travel. Overall, the results support the value of student-led conferences within undergraduate psychiatry, and emphasise the importance of accessibility in maximising their impact and reach.

